# Supplementary material for: A dry lunar mantle reservoir for young mare basalts of Chang’e-5
Source: Nature. 2021 Oct 19;600(7887):49–53. doi: 10.1038/s41586-021-04107-9 (PMC8636271; doi:10.1038/s41586-021-04107-9)
Supplement: Supplementary file 1 — Supplementary Figs. 1–6 and references. [file 41586_2021_4107_MOESM1_ESM.pdf]

nature portfolio

https://doi.org/10.1038/s41586-021-04107-9

Supplementary information

A dry lunar mantle reservoir for young mare basalts of Chang'e-5

In the format provided by the authors and unedited

Supplementary information

Supplementary figures.

Fig. S1. Petrography of CE5 ilmenite-hosted melt inclusions.
Fig. S2. Occurrence of apatite from CE5 basalt clasts.
Fig. S3. CE5 pyroxene quadrilateral diagram.
Fig. S4. Ternary diagram of plagioclase and the variation of the Fa-contents of olivine from CE5 basalt clasts.
Fig. S5. Major element compositions of ilmenite-hosted melt inclusions.
Fig. S6. Apatite ternary plot from CE5 basalt clasts.

Supplementary tables.

Table S1. Modal abundance of apatite in the CE5 basalt clasts.
Table S2. EPMA results of the CE5 basalt clasts.
Table S3. NanoSIMS results of the standards and silicates of the CE5 basalt clasts.
Table S4. Water abundances and hydrogen isotope compositions of ilmenite-hosted melt inclusions and apatite with correction for spallation effects.
Table S5. Summary of water abundances and hydrogen isotope compositions of apatite and melt inclusions from Apollo samples in the literature.

References in the Supplementary Tables and Figures

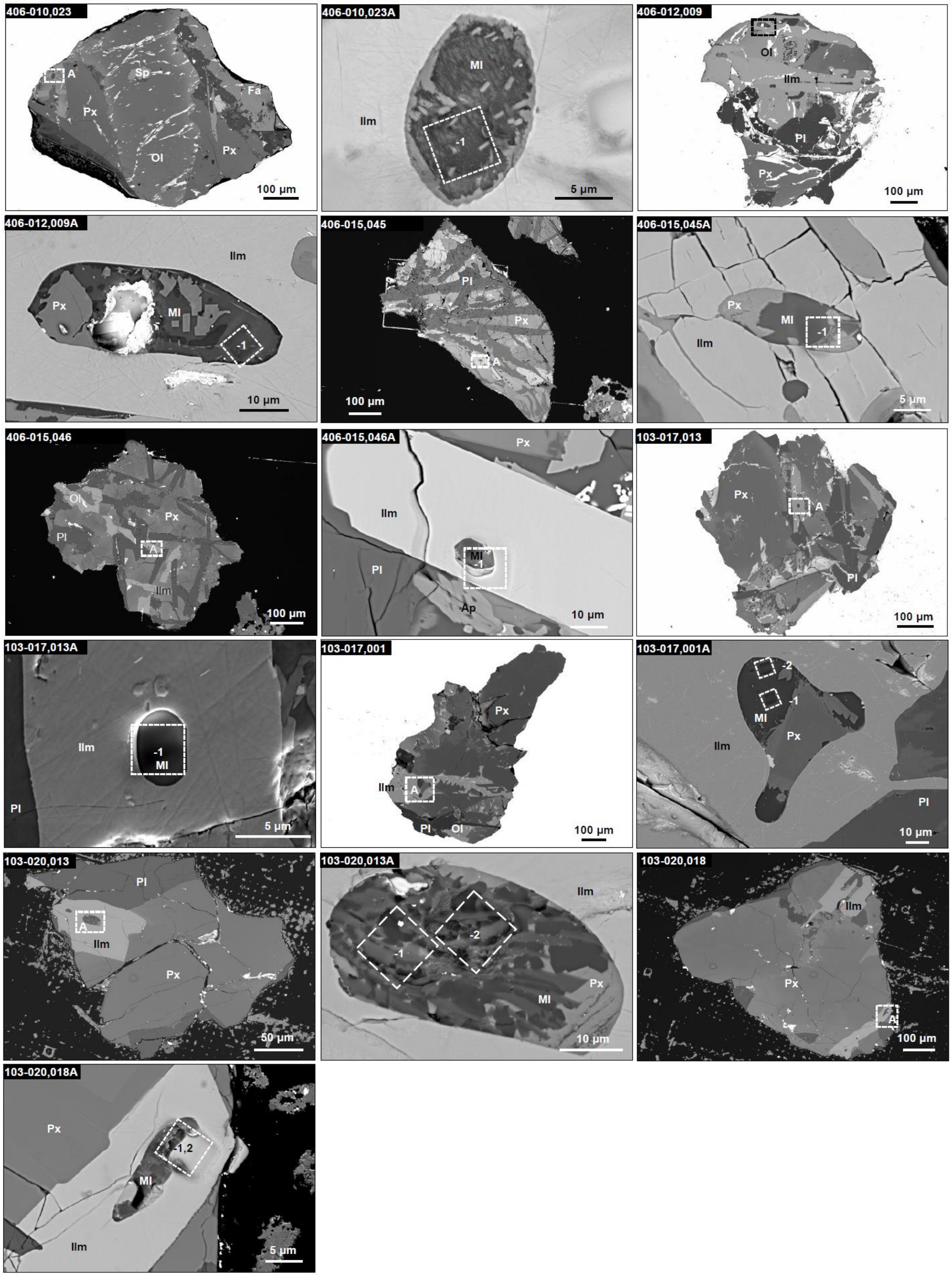

Figure S1 | Occurrences of the ilmenite-hosted melt inclusions from CE5 basalt clasts. Phase abbreviation is identical to Figure 1 in the main text.

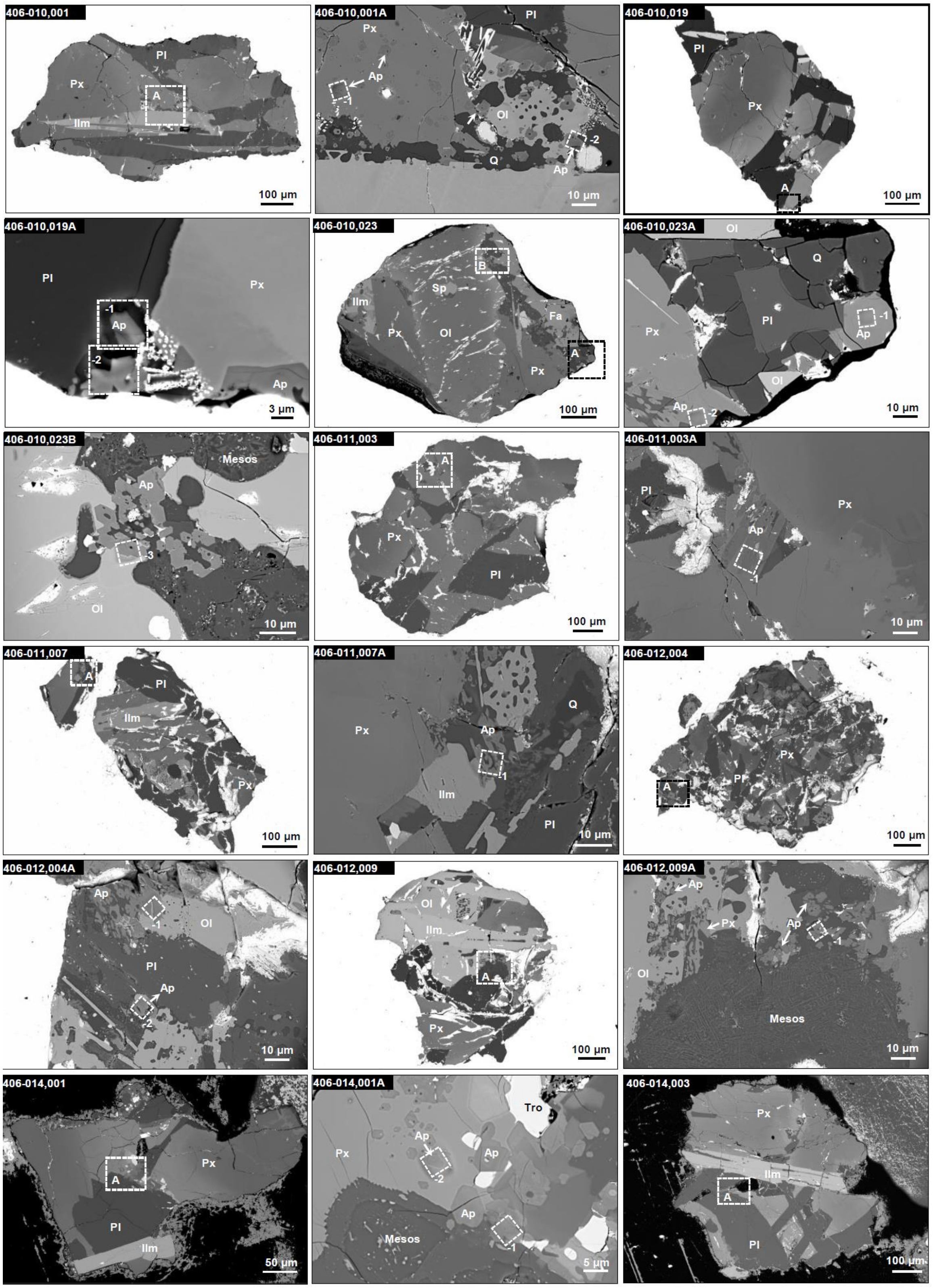

Figure S2 | Occurrences of apatite from CE5 basalt clasts. Phase abbreviation is identical to Figure 1 in the main text.

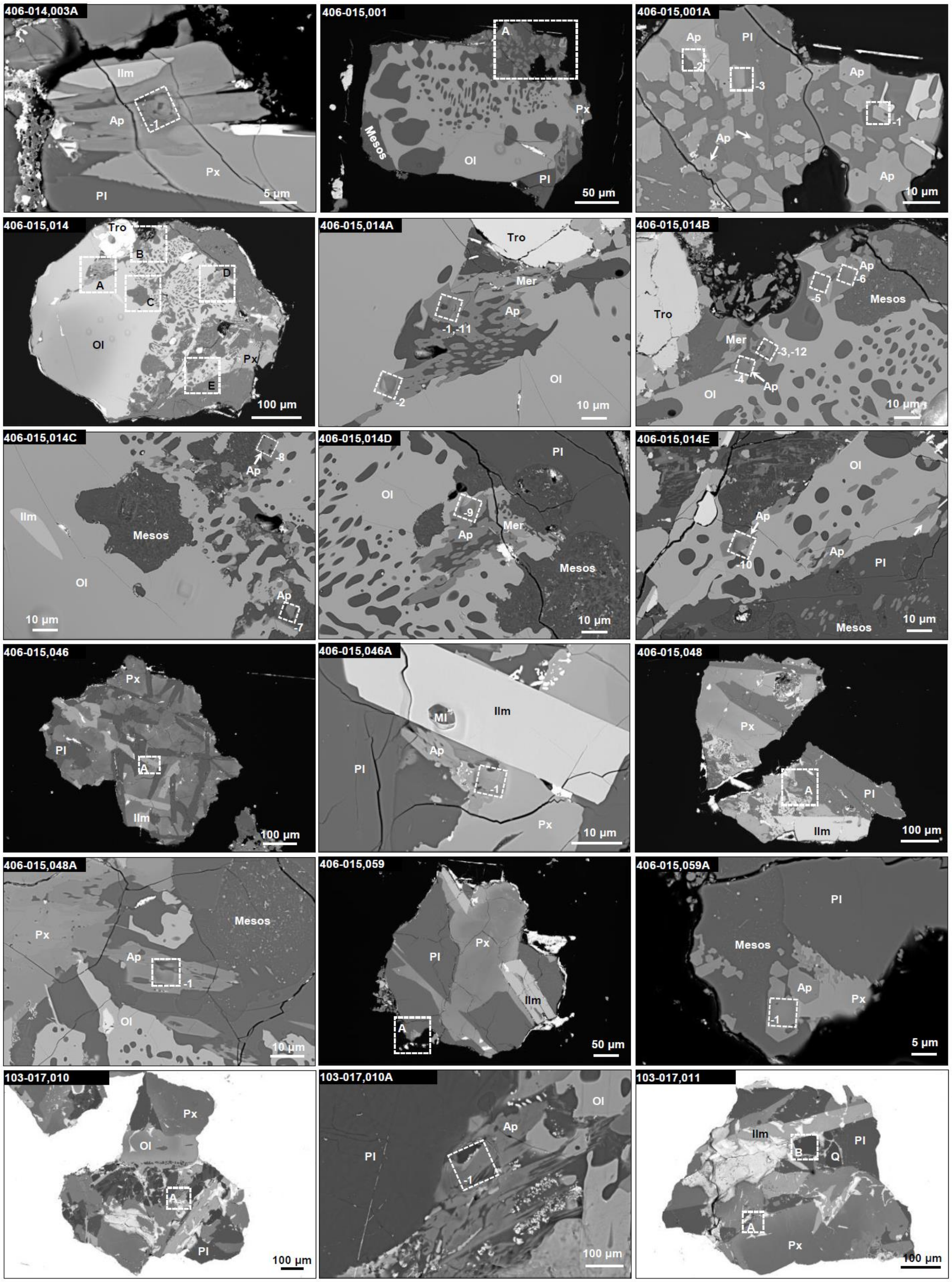

Figure S2 | continued.

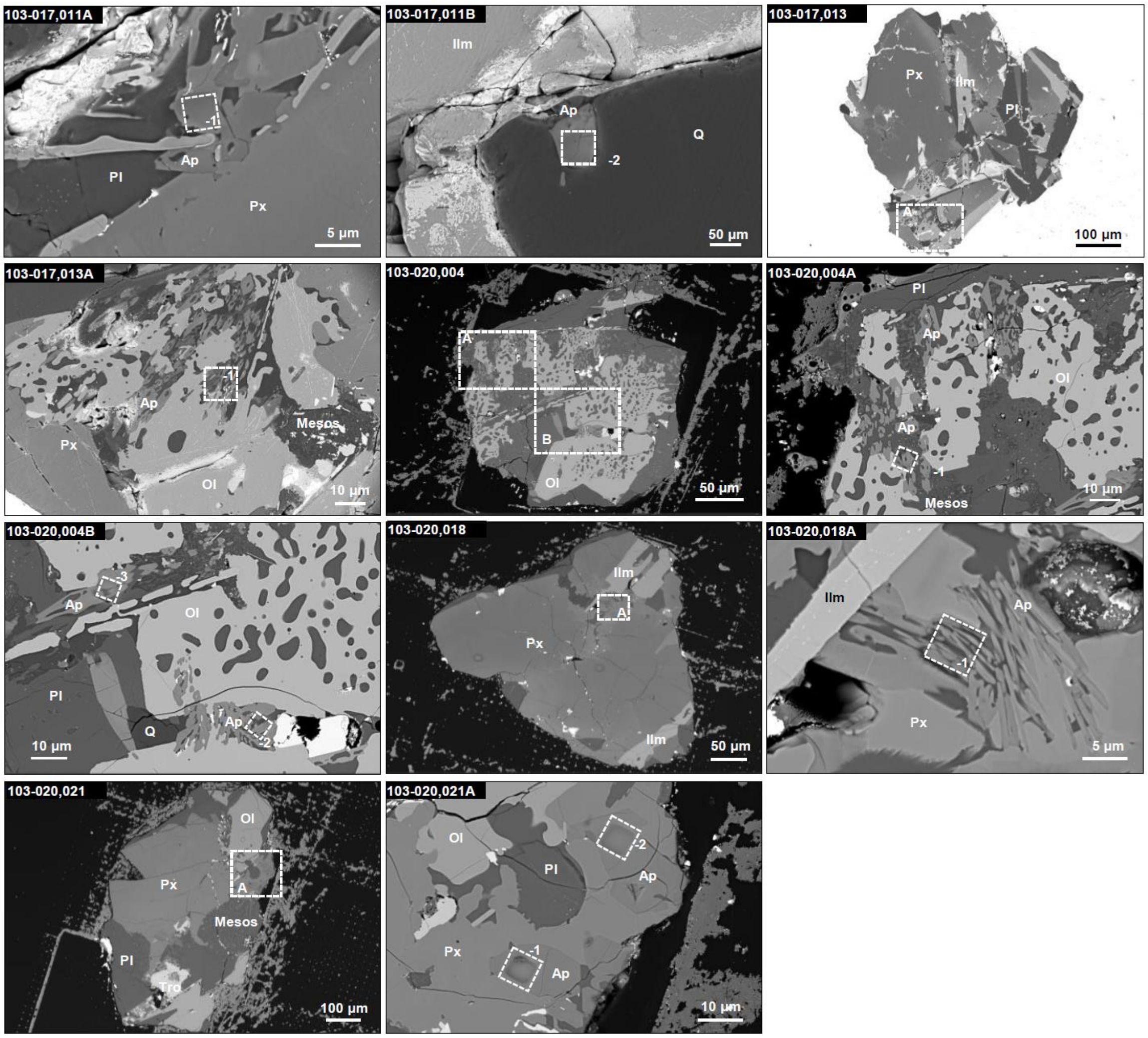

Figure S2 | continued.

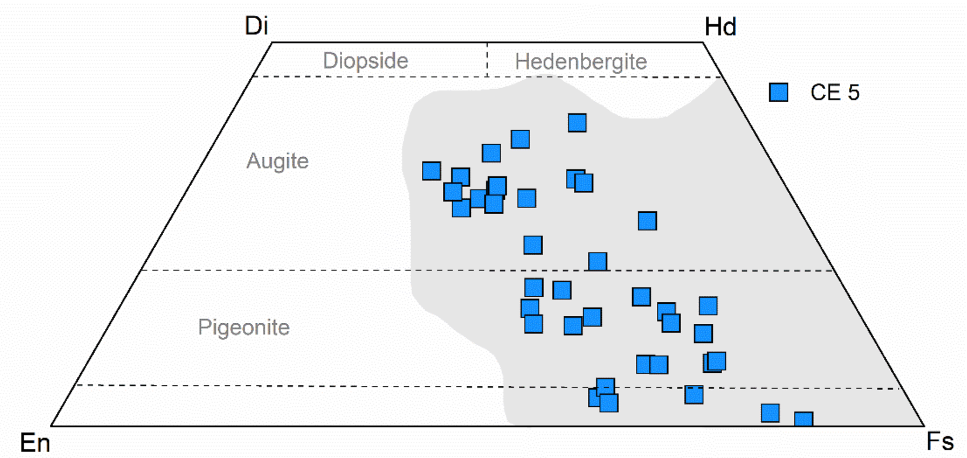

Figure S3 | Pyroxene quadrilateral diagram of the CE5 basalt clasts compared with those of other CE5 basalt clasts reported by ref. [1] (grey hatched region).

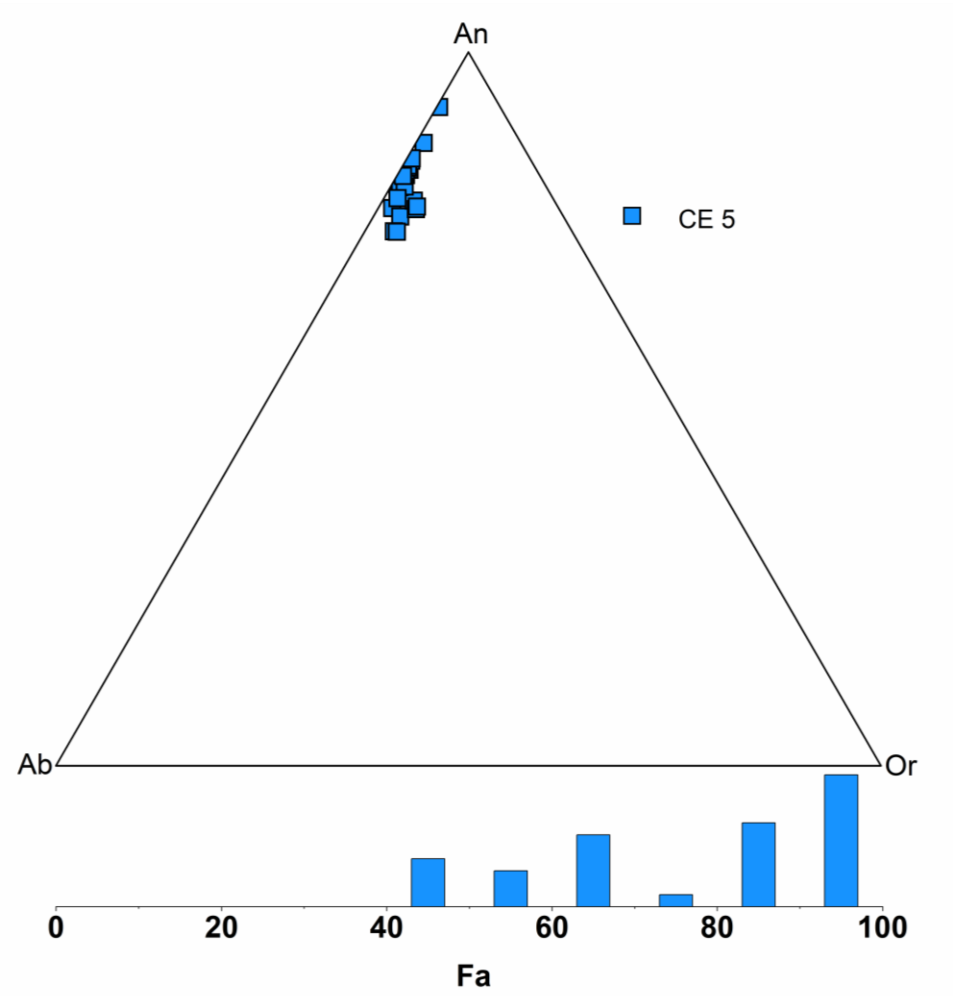

Figure S4 | Ternary diagram of plagioclase and the variation of the Fa-contents of olivine from CE5 basalt clasts.

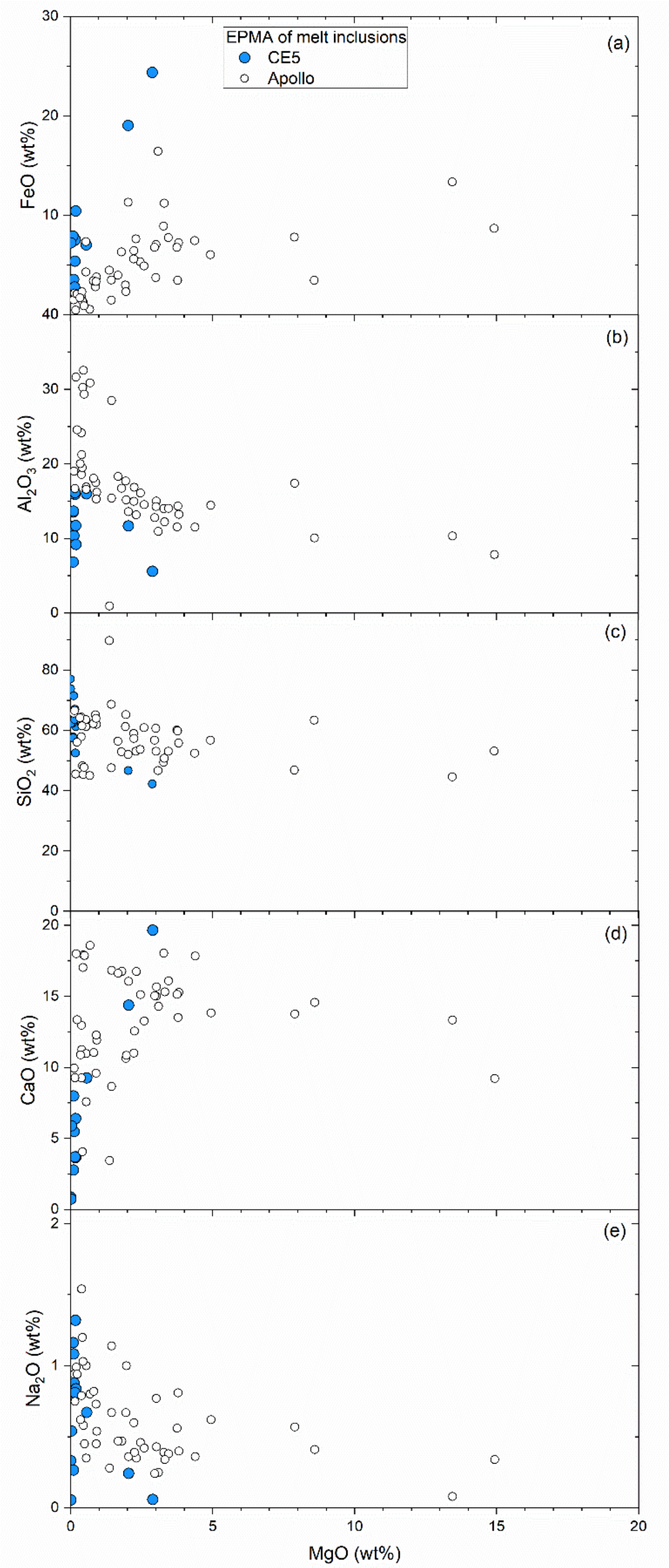

Figure S5 | Major-element compositions of the melt inclusions in ilmenite from the CE5 basalt clasts, comparing with those enclosed in olivine and pyroxene from Apollo mare basalts [2-4].

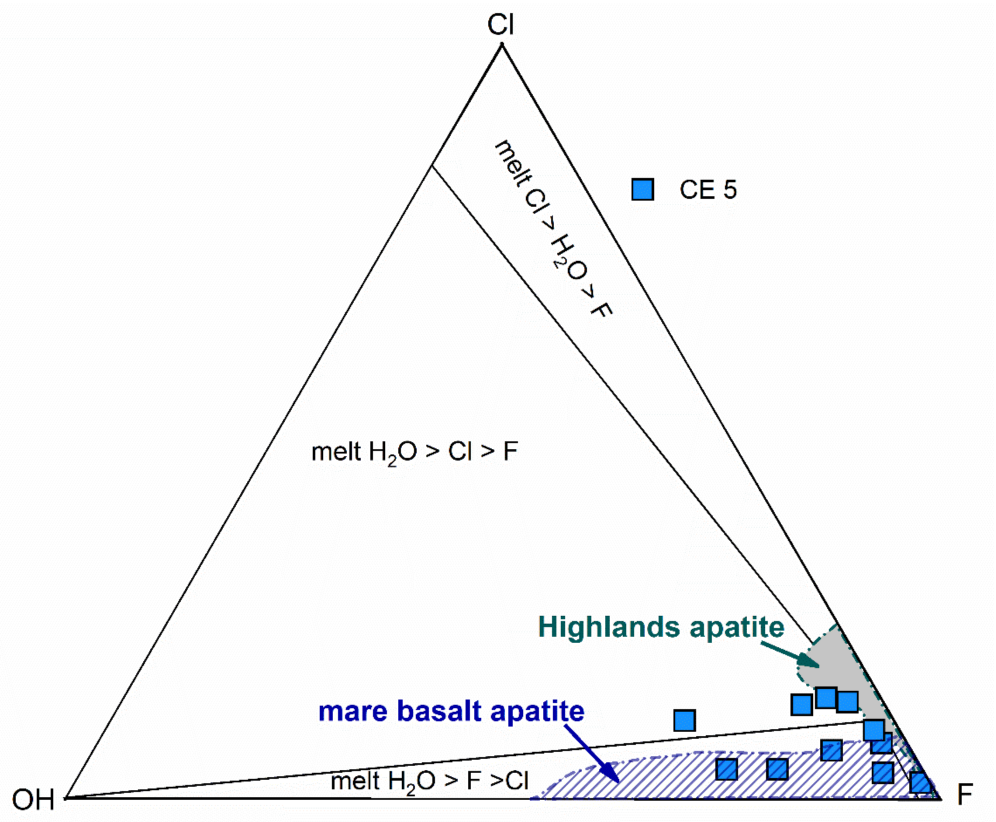

Figgure S6 | The ternary atomic plot of apatite from CE5 basalt clasts (Table S2). Cl and F were measured with EPMA, and OH was calculated based on the stoichiometry of apatite. Fields for apatite composition from Apollo mare basalts and highlands are from literatures[5-10].

References

1. Tian, H.-C., et al. A non-KREEP origin for the Chang'E-5 basalts in the Procellarum KREEP Terrane. *Submitted to Nature* (2021).
2. Stephant, A., et al. The hydrogen isotopic composition of lunar melt inclusions: An interplay of complex magmatic and secondary processes. *Geochim Cosmochim Ac* **284**,196-221 (2020).
3. Hauri, E.H., et al. Water in the Moon's interior: Truth and consequences. *Earth Planet Sc Lett* **409**,252-264 (2015).
4. Hauri, E.H., et al. High pre-eruptive water contents preserved in lunar melt inclusions. *Science* **333**,213-5 (2011).
5. Tartèse, R., et al. The abundance, distribution, and isotopic composition of Hydrogen in the Moon as revealed by basaltic lunar samples: Implications for the volatile inventory of the Moon. *Geochim Cosmochim Ac* **122**,58-74 (2013).
6. Tartese, R., et al. H and Cl isotope systematics of apatite in brecciated lunar meteorites Northwest Africa 4472, Northwest Africa 773, Sayh al Uhaymir 169, and Kalahari 009. *Meteorit Planet Sci* **49**,2266-2289 (2014).
7. McCubbin, F.M., et al. Nominally hydrous magmatism on the Moon. *Proc Natl Acad Sci U S A* **107**,11223-8 (2010).
8. Barnes, J.J., et al. The origin of water in the primitive Moon as revealed by the lunar highlands samples. *Earth Planet Sc Lett* **390**,244-252 (2014).
9. Barnes, J.J., et al. Multiple reservoirs of volatiles in the Moon revealed by the isotopic composition of chlorine in lunar basalts. *Geochim Cosmochim Ac* **266**,144-162 (2019).
10. Boyce, J.W., et al. The chlorine isotope fingerprint of the lunar magma ocean. *Sci Adv* **1**,e1500380 (2015).
11. Barnes, J.J., et al. Accurate and precise measurements of the D/H ratio and hydroxyl content in lunar apatites using NanoSIMS. *Chem Geol* **337**,48-55 (2013).
12. Treiman, A.H., et al. D-poor hydrogen in lunar mare basalts assimilated from lunar regolith. *Am Mineral* **101**,1596-1603 (2016).
13. Greenwood, J.P., et al. Hydrogen isotope ratios in lunar rocks indicate delivery of cometary water to the Moon. *Nat Geosci* **4**,79-82 (2011).
14. Singer, J.A., et al. Evidence for the solar wind in lunar magmas: A study of slowly cooled samples of the Apollo 12 olivine basalt suite. *Geochem J* **51**,95-104 (2017).
15. Pernet-Fisher, J.F., et al. Estimating the lunar mantle water budget from phosphates: Complications associated with silicate-liquid-immiscibility. *Geochim Cosmochim Ac* **144**,326-341 (2014).
16. Tartèse, R., et al. Apatites in lunar KREEP basalts: The missing link to understanding the H isotope systematics of the Moon. *Geology* **42**,363-366 (2014).
